# “IoT-based evaluation of photovoltaic modules enhanced by different reflector materials”

**DOI:** 10.1038/s41598-026-49258-9

**Published:** 2026-05-01

**Authors:** Ahmed Mohamed Abosrea Abdelaziz, Taha Abdelfattah Mohammed Abdelwahab, Ibrahim Seif Ahmed El-Soaly

**Affiliations:** College of Agricultural Engineering, Al-Azhar University, Cairo, Egypt

**Keywords:** Photovoltaic performance, Reflector-assisted solar modules, IoT-based monitoring, Data acquisition system, Optical concentration, Reflector materials, Low-cost PV optimization, Real-time performance analysis, Energy science and technology, Engineering, Materials science

## Abstract

This study presents an integrated low-cost IoT-based evaluation framework assessment of photovoltaic (PV) modules enhanced using low-cost planar reflectors, supported by a custom IoT-based data logging system for real-time performance monitoring. Four identical 50 W mono-crystalline modules were deployed under outdoor conditions in Cairo, Egypt: one reference module and three modules equipped with aluminum foil, galvanized steel, and mirror reflectors. A dedicated ESP32-based monitoring unit was developed to continuously record voltage, current, electrical power, and module temperature with high temporal resolution. The results demonstrate that all reflector materials improved energy generation, with performance strongly dependent on reflector inclination. A 30° reflector angle yielded the highest overall enhancement, where the mirror reflector achieved a maximum increase of 21.2% in daily energy yield relative to the reference module. This improvement, however, was accompanied by an approximate 7 °C rise in module temperature, indicating a clear trade-off between optical gain and thermal loading. Galvanized steel reflectors offered moderate energy enhancement with reduced heating, while aluminum foil reflectors produced smaller gains but remain attractive due to their minimal cost and high availability. Overall, the findings confirm the suitability of simple reflector surfaces for boosting PV output in regions with high solar irradiance, and they validate the accuracy and robustness of the developed IoT-based monitoring system for continuous field evaluation.

## Introduction

 The global demand for clean and sustainable energy has accelerated the adoption of photovoltaic (PV) technology as a reliable renewable energy source^1^. Photovoltaic modules, however, remain limited by two major challenges: their output is restricted by the incident of solar irradiance, and their efficiency decreases as surface temperature rises^2,3^. These challenges are particularly pronounced in hot climate zones such as the Middle East and North Africa, where solar potential is abundant, but module overheating reduces conversion efficiency^4,5^. Photovoltaic modules are primarily characterized by their current, voltage (I–V) and power, voltage (P–V) relationships, which are strongly influenced by solar irradiance and module temperature^2^. Under Standard Test Conditions (STC), the short-circuit current (Isc) scales almost linearly with irradiance, while the open-circuit voltage (Voc) decreases with temperature at a rate of approximately − 0.3 to − 0.4% /°C. Several stdies^6,7^ have emphasized the importance of accurate measurement of both irradiance and temperature to evaluate real-world PV performance. These parameters form the foundation for analyzing the effects of enhancement techniques and monitoring systems. To enhance solar capture, researchers have proposed optical reflectors as a low cost means of redirecting additional irradiance onto PV surfaces^8^. Flat reflectors, V-trough concentrators, and parabolic designs have all shown potential for boosting energy yield^9,10^. Yet, this optical gain is often counterbalanced by increased thermal stress on the modules, leading to higher operating temperatures and efficiency losses^11,12^. The dual effect of reflectors enhancing irradiance while aggravating thermal conditions requires systematic performance evaluation under real outdoor conditions. Optical reflectors have been widely studied as a cost-effective method to increase the incident irradiance on PV modules. Flat mirrors, aluminum sheets, and V-trough concentrators are among the most common designs. For instance^13^, demonstrated that V-trough reflectors can increase the incident irradiance, leading to a proportional increase in current output. Similarly^14^, evaluated planar side reflectors and reported gains of 20–30% in power output under clear-sky conditions. However, multiple studies have also documented the thermal penalty associated with reflector systems. Increased irradiance leads to elevated surface temperatures, which in turn reduce module efficiency^15^. reported efficiency losses of up to 0.5% per °C rise in temperature, highlighting the trade-off between optical gain and thermal degradation. Research on cooling techniques (water spraying, passive heat sinks) has been suggested as a mitigation strategy but remains underexplored in conjunction with reflectors. Overall, reflector-assisted PV systems demonstrate clear potential for energy yield enhancement, but their real-world viability is contingent on managing thermal effects and continuously monitoring performance. In parallel, accurate monitoring of PV performance has become essential for both research and deployment. Conventional data acquisition systems offer high precision but are cost prohibitive, limiting their use in small scale or experimental setups. The emergence of Internet of Things (IoT) technologies provides an alternative pathway: low-cost microcontroller-based data loggers equipped with voltage, current, and temperature sensors can collect, store, and transmit data in real time to cloud platforms^16–18^. Such systems enable high-resolution monitoring and democratize access to performance data, particularly in resource-constrained settings. The development of low-cost data acquisition systems has gained considerable attention in recent years, particularly for monitoring photovoltaic (PV) systems. Several studies have focused on integrating IoT and microcontroller-based solutions to improve real time monitoring and data accessibility^19^. For instance, an ESP32 based system was introduced to capture voltage, current, and temperature while simultaneously transmitting the data to a Thing Speak server and displaying it on an LCD module, thus combining both local and remote monitoring features^20^. Similarly, a study presented a cost effective IoT enabled DAQ system, designed to offer real time measurements suitable for small to medium scale PV applications, highlighting affordability as a major advantage^21^. Energy efficiency has also been addressed by other researchers. An ultra-low power data logger employing ESP32-S2 with deep sleep mode was proposed, capable of logging data onto an SD card while hosting a lightweight web server for real-time visualization^22^. Additionally, a proof-of-concept open-source data logger demonstrated the potential of Wi-Fi-based monitoring solutions in broader engineering applications, validating the flexibility of low-cost microcontroller platforms for telemetry systems^23^. On the smaller scale, a micro-PV monitoring system showcased the use of IoT based approaches to record real time voltage in small scale solar energy generation, further reinforcing the adaptability of such systems in resource limited contexts^24^. While these systems demonstrate significant progress in PV monitoring technologies, several limitations remain. Most existing designs either prioritize low cost or power efficiency but lack integrated multi parameter monitoring, particularly the inclusion of panel-surface temperature sensors and onboard filtering of electrical signals^25^. Moreover, very few solutions provide a combination of local visualization, online data transmission, and advanced analytical support. These gaps highlight the need for a more versatile, robust, and scalable data logger, which is the focus of the present work. Despite extensive work on reflector-assisted PV modules and on IoT-based monitoring systems, the two domains have rarely been integrated. Previous reflector studies often relied on limited manual measurements, overlooking transient fluctuations and thermal dynamics. Conversely, existing IoT monitoring systems typically evaluate standalone PV modules without performance enhancement techniques. This lack of integration leaves a knowledge gap in understanding the combined effects of optical enhancement and real time digital monitoring. This study addresses this gap by designing and experimentally validating a low cost IoT based data logger for evaluating the performance of reflector assisted PV modules. The main contributions are: (1) Development of a reliable ESP32 based logging system that measures electrical (voltage, current, power) and thermal (module and ambient temperature) parameters with cloud integration. (2) Experimental investigation of a 50 W mono-crystalline PV module under reflector and non-reflector conditions, quantifying the balance between irradiance gain and temperature-induced losses. (3) Provision of a comparative dataset that highlights the benefits and limitations of reflector-assisted PV modules when monitored with affordable IoT tools. By bridging optical enhancement with digital monitoring, the proposed approach advances both PV performance evaluation and the accessibility of monitoring technologies, offering practical insights for deployment in developing regions.

## Materials and methods

### Photovoltaic modules

The experimental setup employed four identical mono-crystalline PV modules, each rated at 50 W (CY50-36P). The electrical and thermal characteristics of the 50 W mono-crystalline PV module used in this work are presented in Table 1. These modules were selected as representative units for small-scale PV systems typically used in research and field applications in Egypt.

**Table 1:** The used PV system specifications.

Parameter	Rated Value	Remarks / Test Condition
Maximum Power (Pmax)	50	At Standard Test Conditions (STC): 1000 W/m², AM1.5, 25 °C
Open-Circuit Voltage (VOC)	22.32	Measured at STC
Short-Circuit Current (ISC)	3.01	Measured at STC
Voltage at Maximum Power (VMP)	18.00	—
Current at Maximum Power (IMP)	2.78	—
Module Dimensions	730 × 540 × 25 mm	Including aluminum frame

**Fig. 1:**
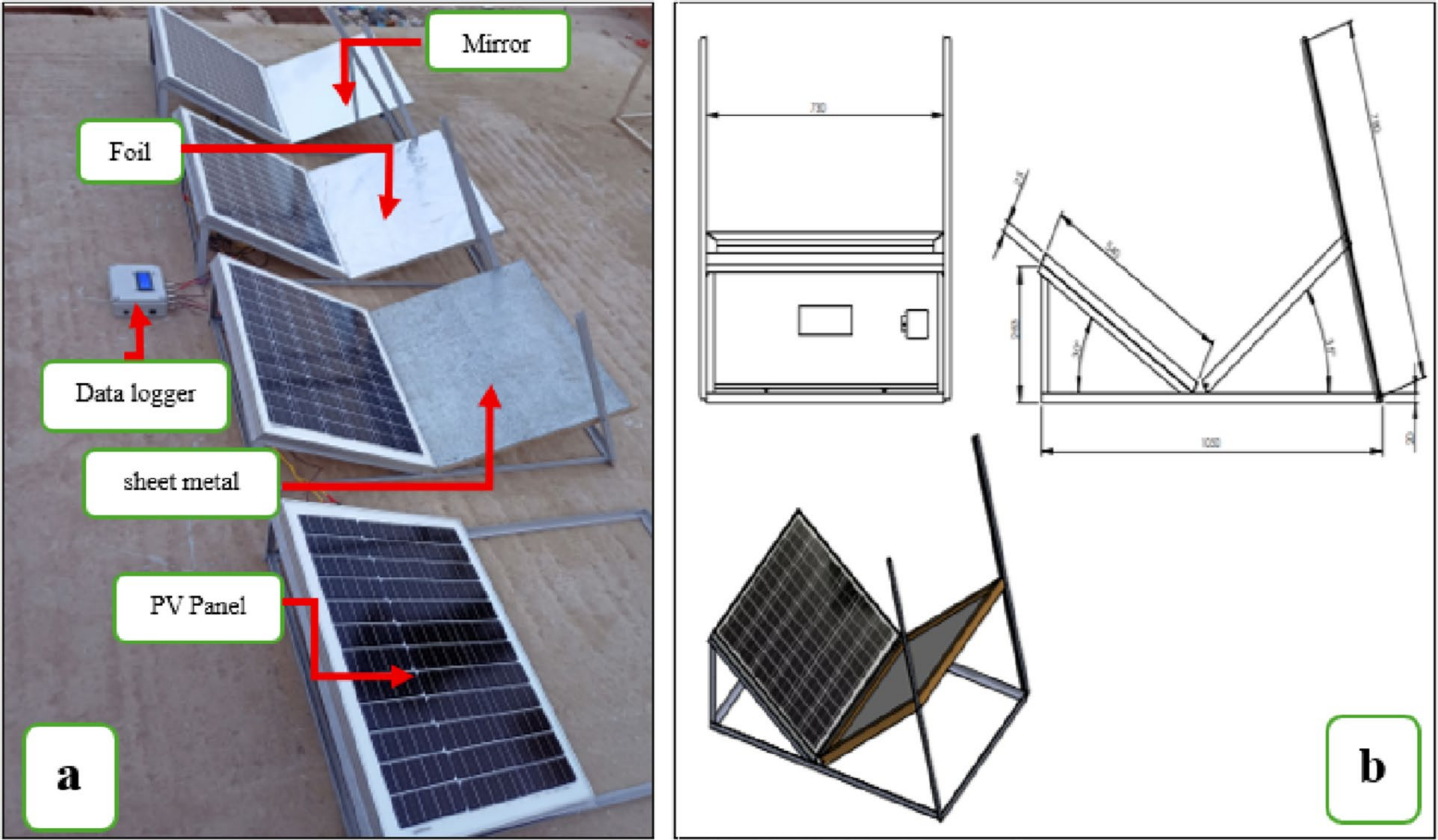
(**a**) Experimental Validation, (**b**) Setup Design.

These experiments were carried out in Cairo, Egypt, considering the local climate. Egypt is known for its desert climate. The weather is hot and dry most of the time. The coordinates of Egypt’s capital, Cairo, are 30.04167° N, 31.23528° E. Cairo receives 4.7 kWh/m2/day of solar energy, and the average annual temperature is 22.1 °C. On average, the yearly wind speed is 4.02 m/s, and the humidity is 55%. Details of the outside circumstances in August 2025, the day of the testing, are displayed in Fig. 1. August is a bright month with high temperatures, moderate breezes, and low humidity on most days. The modules had a 30° inclination and were oriented southward at Cairo latitude.

### Reflector design and configuration

To investigate the effect of different reflector materials on PV performance, three types of planar reflectors were tested. Each reflector had the same dimensions as the PV module (730 × 540 mm) to ensure full redirection of incident sunlight. The reflector materials were: (1) Galvanized sheet metal, (2) Glass mirrors, (3) Aluminum foil. Three identical PV modules were employed in this experiment, each equipped with a front-mounted reflector installed at a fixed tilt angle of **20°**,** 30°**,** and 40°** relative to the module plane. During the experimental setup, careful attention was given to spatial separation and geometric alignment between modules to minimize potential cross-illumination effects. The modules were installed with sufficient lateral spacing to reduce the likelihood of reflected radiation from one configuration reaching the reference module. Additionally, all reflectors were oriented toward their corresponding module surfaces, with inclination angles designed to direct reflected radiation toward the active area of the enhanced module rather than laterally toward neighboring units. This setup was designed to investigate the influence of different reflector tilt angles on the electrical performance and energy yield of the PV modules. The fourth PV module, without any reflector (control), served as the reference module.

### Instruments used

A customized data acquisition system was developed to continuously monitor the electrical and thermal behavior of the photovoltaic modules throughout the experiment. The system was designed to record key parameters voltage, current, power, and temperature in real time, ensuring precise performance tracking under varying irradiance and environmental conditions. Small variations consistent with typical ± 5% manufacturing tolerance were observed. To ensure fair comparison during outdoor testing, a normalization factor was introduced in the data acquisition system. Each module’s measured electrical output was corrected using its respective baseline calibration coefficient derived from the pre-characterization stage. This correction minimized bias due to intrinsic module variation and ensured that performance differences observed during the experiment were primarily attributable to reflector effects rather than manufacturing tolerances. It provided both local data storage and remote accessibility, enabling high resolution monitoring and post analysis of PV behavior without manual intervention.

#### Sensors

The developed data acquisition unit was designed to monitor the electrical and thermal performance of photovoltaic modules in real time. Current measurements were performed using the ACS712 current sensor (5 A range), providing high sensitivity and electrical isolation^26^. The sensor was calibrated against a laboratory-grade multimeter over the 0–5 A range, achieving an average measurement error of ± 2% and incorporating a ± 0.2 A dead-band for noise rejection. Panel voltage was monitored through a precision voltage divider circuit, ensuring safe interfacing with the microcontroller’s analog-to-digital converter. All measurements were performed under real operating conditions using a fixed resistive load of 6.8 Ω (50 W), selected to approximate the nominal operating region of the 50 W mono-crystalline modules under standard test conditions. A precision shunt resistor was used for current measurement, while voltage was measured across the load terminals. Electrical power was calculated as $\:P\:=\:V\:\times\:\:I$. Thermal measurements were obtained using four DS18B20 digital temperature sensors mounted on the rear side (backsheet) of each module for accurate temperature tracking^27^. The sensors were individually calibrated with a reference thermometer, resulting in a ± 0.5 °C accuracy at a sampling frequency of 1 Hz. Each sensor was connected via a 1-Wire communication bus with a 4.7 kΩ pull-up resistor for reliable data transfer. The core of the system was an ESP32 microcontroller, selected for its dual-core processing capability and built-in Wi-Fi, enabling real-time wireless data transmission^28^. To accommodate multiple analog inputs, an ADC multiplexer module was integrated^29^, while a step-down DC–DC converter ensured stable voltage regulation and noise suppression^30^. User interaction was facilitated through an LCD2004 I2C display, which provided real-time readouts of voltage, current, power, and temperature parameters^31^. The enclosure also included a side ON/OFF power switch and a female DC charging port for convenient system maintenance. Power was supplied by a 12 V, 5000 mAh lithium-ion battery pack, allowing extended autonomous operation under field conditions.

**Fig. 2:**
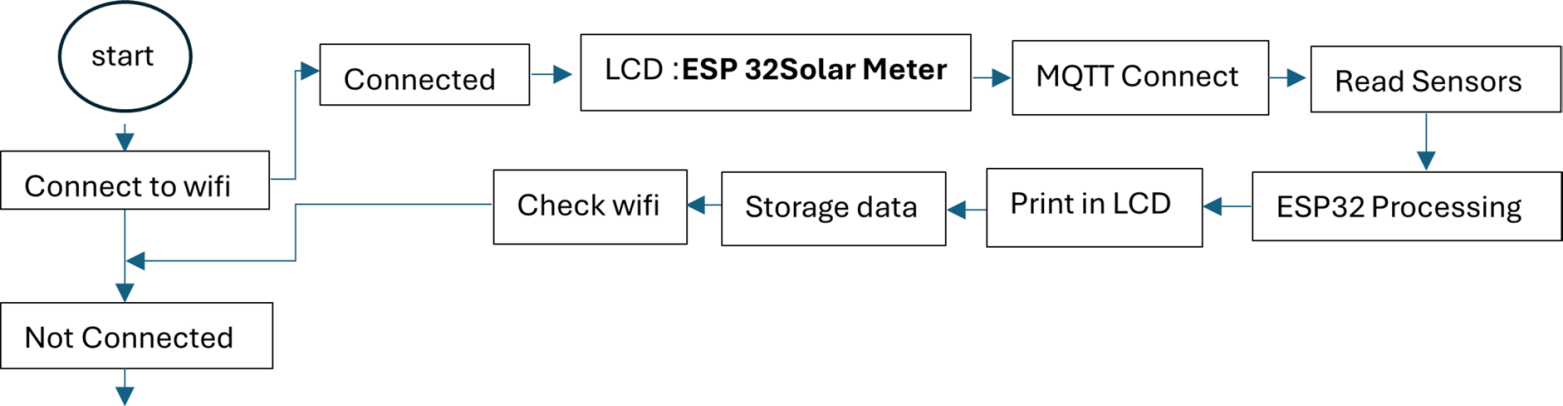
Block diagram of the system.

**Fig. 3:**
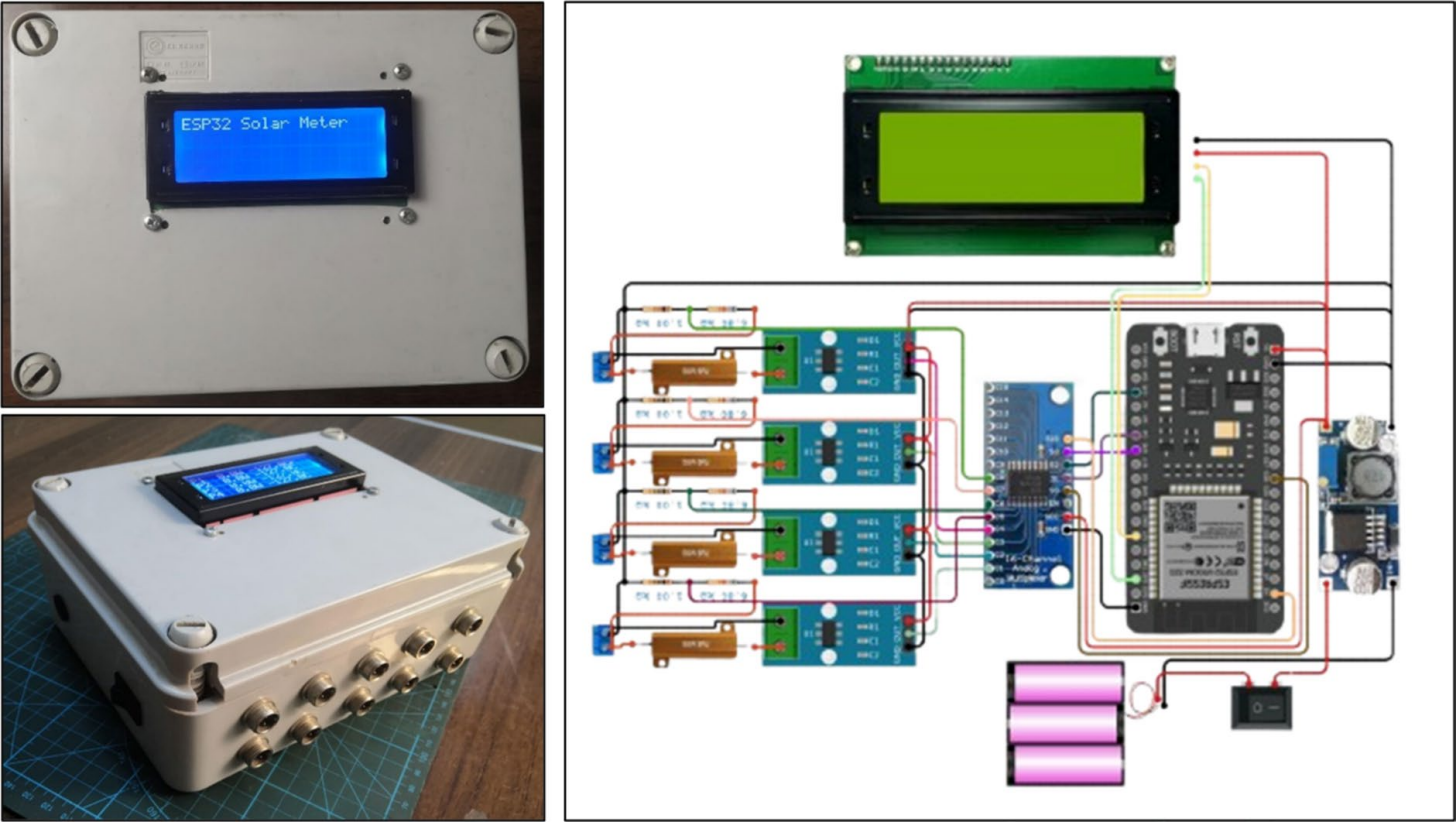
Circuit design of the Solar Data Logger System.

#### Software design

The software component of the monitoring system was developed using Visual Studio Code with the ESP32 programming environment. The program architecture was structured into four functional layers to ensure efficient data handling and system reliability. The data acquisition layer continuously collects voltage, current, and temperature readings from the connected sensors at fixed intervals of five minutes, performing real-time calibration and noise filtering to enhance signal accuracy. The data processing layer computes the instantaneous electrical power from the measured parameters, integrates the power values over time to estimate the total energy yield, and applies temperature correction factors to account for variations in module efficiency. The data visualization layer provides immediate feedback through an LCD2004 I2C display, presenting live values of voltage, current, power, and temperature. Additionally, the processed data are transmitted through the ESP32 integrated Wi-Fi module to a web-based dashboard, enabling real-time remote monitoring and data analysis.

## Results and discussions

On 15 August 2025, a field experiment was conducted in Cairo, Egypt under clear-sky summer conditions to evaluate the performance of low-cost bilateral flat reflectors applied to fixed-tilt monocrystalline silicon photovoltaic modules. Four identical 50 W modules were mounted side by side on a south facing structure at a typical Cairo tilt angle of ~ 30°. Three of the modules were equipped with bilateral reflectors made of commercial mirror (ρ ≈ 92–94%), galvanized steel sheet (ρ ≈ 70–75%), and ordinary aluminum foil (ρ ≈ 65–70% under field conditions), each pair inclined at 20°, 30°, and 40° from horizontal respectively, while the fourth module served as an reference Module. Back-surface module temperature, maximum-power-point voltage (Vmp), and instantaneous power output (Pmp) were continuously logged every 5-minute intervals from before sunrise (~ 05:50) until after sunset (~ 18:50) our data logger. Ambient temperature ranged from 28 °C in the early morning to a peak of 38 °C around 14:00, providing ideal conditions to quantify both the optical enhancement and the associated thermal penalty introduced by the reflector systems in a hot, high-insolation urban desert environment.

### Voltage and current profiles

To assess the electrical behavior of the photovoltaic modules under reflector-assisted operation, the diurnal voltage–time profiles were examined across all reflector materials and inclination angles. Voltage is particularly sensitive to temperature rise and serves as an effective indicator of how optical enhancement interacts with thermal effects throughout the day. The following voltage curves therefore provide a comparative representation of the reference module and the three reflector-assisted modules at 20°, 30°, and 40°, enabling a clear evaluation of the net impact of reflector type and geometry on the module’s electrical response.

**Fig. 4:**
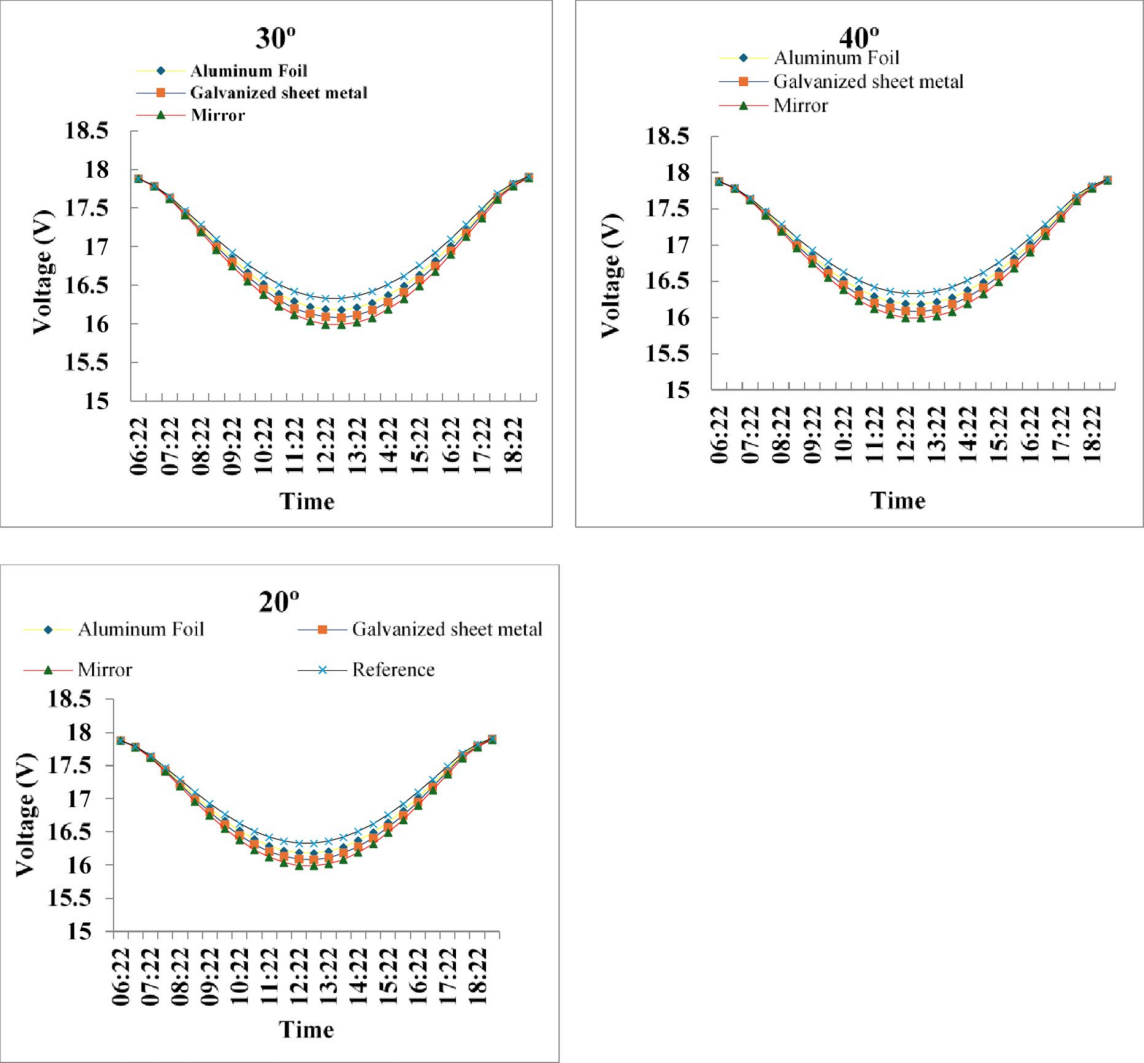
Voltage profiles of PV modules with different reflectors (mirror, galvanized sheet metal, aluminum foil) at 20°, 30°, and 40° on 15 August 2025. The mirror at 30° shows the largest voltage drop due to increased heating.

The voltage – time profiles for reflector inclinations of 20°, 30°, and 40° exhibit the characteristic midday voltage suppression associated with increased cell temperature and enhanced irradiance. Across all configurations, reflector-assisted modules consistently recorded lower voltages than the reference module, with the mirror reflector producing the most pronounced reduction due to its superior reflectivity and corresponding thermal loading. At 20°, the reflected irradiance was only partially aligned with the module surface, resulting in modest voltage deviations. In contrast, the 30° configuration generated the strongest voltage drop near solar noon, indicating optimal geometric alignment between incident and reflected rays and therefore the highest thermal impact. When the angle increased to 40°, the voltage depression remained evident but slightly diminished, reflecting partial misalignment and reduced optical concentration. The relative behavior of the reflector materials was consistent across all angles mirror > galvanized sheet metal > aluminum foil in terms of thermal effect demonstrating that higher reflectivity leads to greater temperature-induced voltage reduction. Overall, the voltage patterns affirm the inverse relationship between irradiance-driven heating and PV voltage, and they highlight 30° as the most effective inclination for maximizing the optical enhancement of flat planar reflectors under outdoor conditions. 

**Fig. 5:**
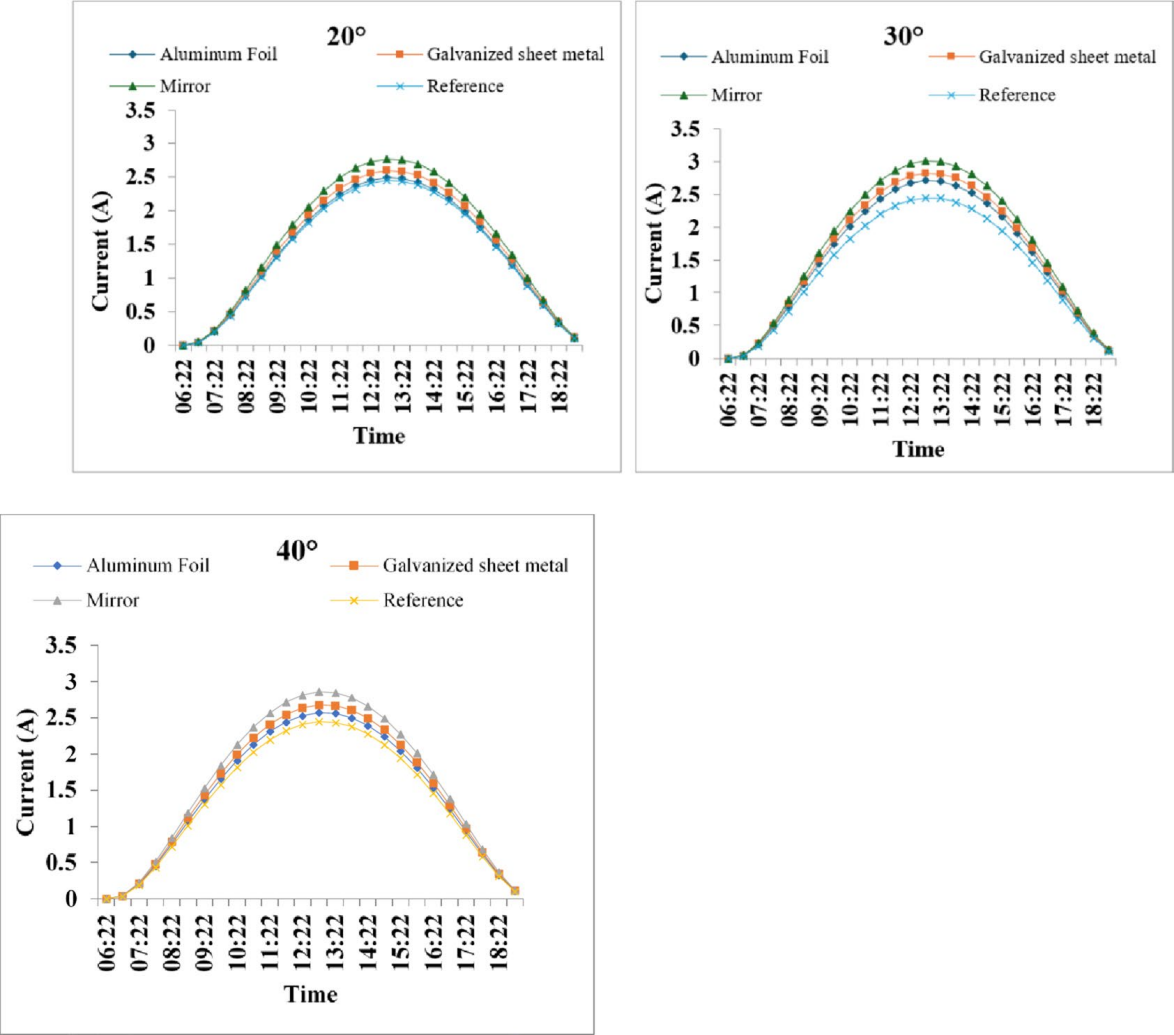
Current profiles of PV modules under different reflectors at 20°, 30°, and 40° on 15 August 2025. The mirror at 30° achieves the highest current.

The experimental results, illustrated in Fig. (5), demonstrate the diurnal variation in short-circuit current (Isc) of a photovoltaic panel under three reflector configurations aluminum foil, galvanized sheet metal, and mirror tested at tilt angles of 20°, 30°, and 40°, alongside a reference panel without any reflector. The mirror consistently achieved the highest peak currents, with a maximum of 3.01 A at 30°, representing a 22.9% enhancement over the reference (2.45 A). This superior performance is attributable to the mirror’s high specular reflectivity, which efficiently redirects diffuse and direct sunlight onto the PV surface. In contrast, aluminum foil and galvanized sheet metal, with lower reflectivity (approximately 85–90% and 60–70%, respectively, based on literature values for similar materials), yielded moderate improvements, peaking at 2.71 A and 2.81 A at 30°.

Notably, the 30° angle produced the highest peaks across all reflectors, suggesting an optimal balance between reflector tilt and solar incidence angle for maximizing reflected flux without excessive shading or optical losses. At 20°, the shallower angle may have resulted in suboptimal reflection geometry, leading to reduced enhancements. At 40°, increased scattering or edge losses could explain the slightly lower peaks compared to 30°.

### Temperature behavior

Module operating temperature is a critical parameter in photovoltaic systems, as every 1 °C rise above 25 °C typically reduces crystalline silicon power output by 0.40–0.45%. The addition of flat reflectors increases incident irradiance but also elevates cell temperature due to higher absorbed energy and slightly reduced convective cooling.

**Fig. 6:**
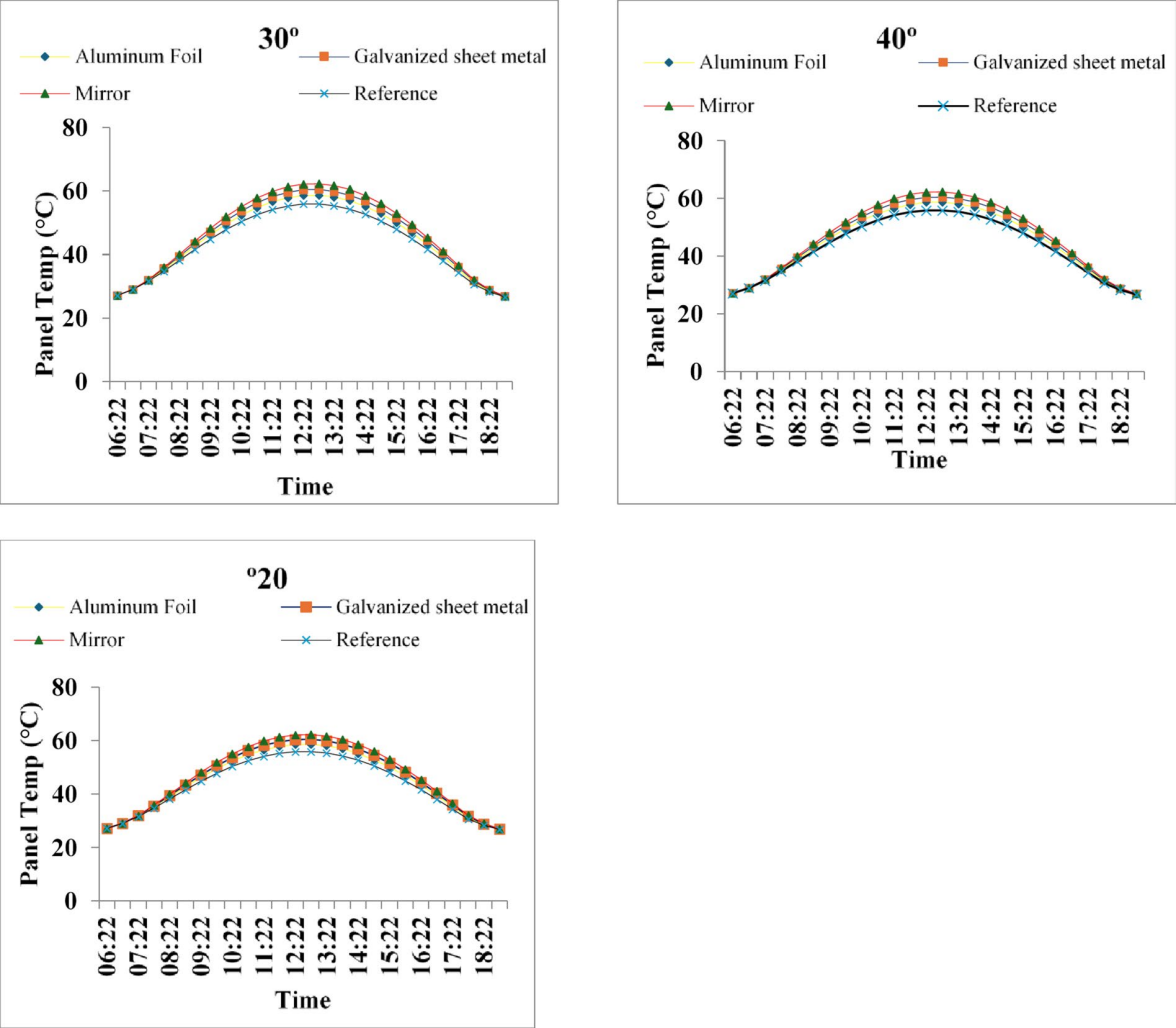
Temperature profiles of PV modules under different reflectors at 20°, 30°, and 40° on 15 August 2025. Reflectors increase temperature by ~ 6–7 °C, with the mirror showing the highest rise.

Measured temperature profiles (Figs. 1, 2 and 3) show that reflectors induce a modest but systematic increase in module temperature. The highest value recorded was 62.3 °C, reached with the mirror reflector at both 30° and 40° inclinations around solar noon, corresponding to a rise of only 6.4 °C above the reference module peak of 55.9 °C. At 20° inclination the mirror peaked at 61.7 °C (+ 5.8 °C), while galvanized sheet and aluminum foil produced progressively smaller elevations owing to their lower reflectivity. Transitioning from 20° to 30° markedly increased midday heating, whereas the further increase to 40° yielded no additional temperature rise for mirror and galvanized reflectors, indicating that most of the geometrically available reflected radiation was already being captured at 30° under high summer solar altitude. The observed temperature increases remained well below the typical NOCT (85 °C) and maximum operating limits of commercial modules, confirming that simple bilateral reflector systems introduce only minor thermal penalties in hot climates when high-reflectivity materials are used.

## Power output

**Fig. 7:**
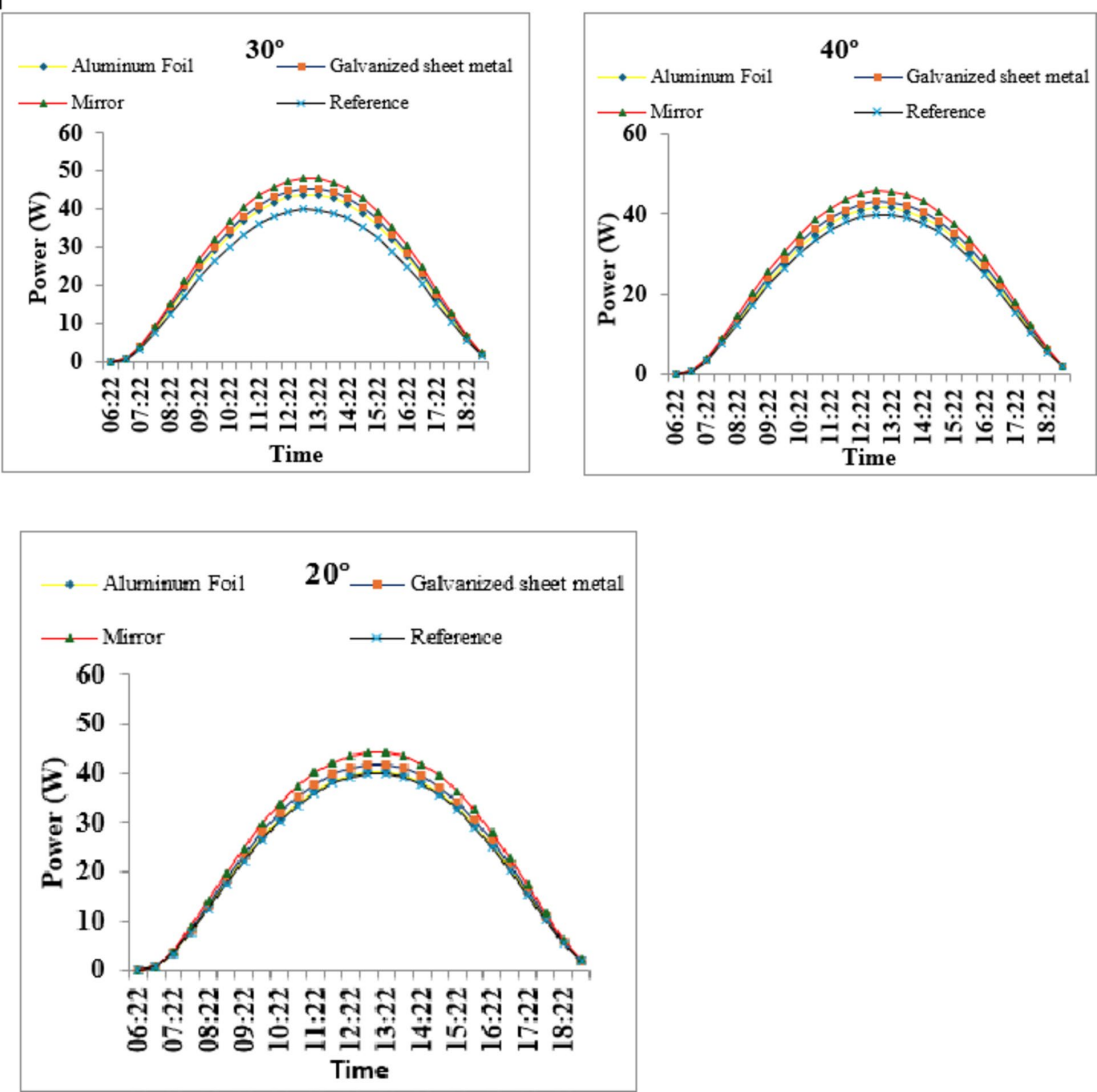
Power output of PV modules with different reflectors at 20°, 30°, and 40° on 15 August 2025. The mirror at 30° provides the highest gain.

To evaluate the net performance impact of reflector integration, the diurnal power–time profiles of the photovoltaic modules were analyzed for all reflector materials and inclination angles. Power output captures the combined effects of increased incident irradiance and the accompanying temperature-induced efficiency losses, making it a direct indicator of the system’s operational energy gain. The following power curves provide a comparative assessment of the reference module and the reflector-assisted modules at 20°, 30°, and 40°, illustrating how reflector type and tilt influence real-time power generation throughout the day.

Power measurements (Figs. 4, 5 and 6) demonstrate substantial electrical gains that significantly outweigh the modest thermal penalty. The highest instantaneous power achieved was 45.68 W with the mirror reflector at 30° inclination (+ 29.3% over the reference), closely followed by 45.60 W at 40° (+ 28.8%). Galvanized sheet configurations delivered 21–22% improvement, while aluminum foil provided 13–22% depending on angle. Raising reflector tilt from 20° to 30° produced the largest incremental gain in peak power, with diminishing returns beyond 30° for high-reflectivity materials, indicating optical collection approaches saturation under high summer sun elevation. Accounting for the simultaneous temperature rise of ~ 6.4 °C (causing ≈ 2.7% power loss at PTC ≈ − 0.42%/°C), the mirror reflector at 30–40° still yields a net instantaneous gain exceeding 25% relative to the reference module. These results confirm that properly designed bilateral mirror reflector systems can deliver energy increases of 20–29% even under hot summer conditions, making them a highly cost-effective enhancement for fixed-tilt PV installations in sunny climates.

### Performance comparison

To clearly illustrate reflector performance, the relative energy improvement compared to the reference panel is presented in Table 2.

**Table 2:** Percentage improvement in daily energy compared to reference.

Reflector Type	20° Angle	30° Angle	40° Angle
Mirror	+ 11.53%	+ 21.23%	+ 15.17%
Galvanized sheet metal	+ 5.02%	+ 14.15%	+ 8.44%
Aluminum Foil	+ 1.14%	+ 9.94%	+ 4.44%

The results demonstrate that reflector angle plays a significant role. A 30° reflector inclination yielded the best overall results, aligning with the optimal tilt of the PV panel itself.

The mirror consistently provided the highest performance gains across all tested angles, achieving improvements of + 11.53% at 20°, + 21.21% at 30°, and + 15.17% at 40°. This clearly demonstrates the mirror’s superior specular reflectivity, which maximizes the redirection of both direct and diffuse irradiance onto the PV panel surface with minimal losses.

The 30° tilt angle emerged as the optimal configuration overall, delivering the largest relative enhancements for every reflector material: +21.21% (mirror), + 14.15% (galvanized sheet metal), and + 9.94% (aluminum foil). This intermediate angle appears to strike the best balance between capturing low-angle sunlight in the morning and afternoon while avoiding excessive optical losses, scattering, or self-shading during peak solar hours.

In comparison, the steeper 40° tilt yielded more moderate gains (+ 15.17% for mirror, + 8.44% for galvanized sheet metal, and + 4.44% for aluminum foil), likely due to reduced effective reflected flux at midday as light is directed beyond the panel edges. At the shallowest 20° angle, benefits were noticeably smaller (+ 11.53% for mirror) and quite limited for the lower-reflectivity materials (+ 5.02% for galvanized sheet metal and only + 1.14% for aluminum foil), suggesting that very low tilts may redirect insufficient light toward the panel or introduce minor shading effects that offset gains.

### Cost analysis

#### Experimental setup and reflector assemblies

The experimental setup consisted of four independent PV frames, each supporting a 50 W mono-crystalline PV module. Three frames were fitted with planar reflectors fabricated from mirror glass, galvanized steel, and aluminum foil, respectively, while the fourth frame served as a reference without any reflector. Each frame allowed precise angle adjustment and independent electrical interfacing with the data logger Table 3.

**Table 3:** Estimated cost of PV modules and reflector assemblies (USD).

Component	Specification	Qty	Unit Price (USD)	Total (USD)
PV module (CY50-36P)	50 W mono-crystalline	4	41.9	167.6
Mechanical frame (adjustable)	Independent steel/aluminum structure	4	11.8	47.2
Mirror reflector	Glass mirror (> 90% reflectivity)	1	4.3	4.3
Galvanized sheet reflector	Steel sheet (~ 75% reflectivity)	1	3.0	3.0
Aluminum foil reflector	Reflective foil	1	1.1	1.1
Electrical wiring	Conductors and connectors	1 set	8.6	8.6
**Total Experimental Setup**	**—**	**—**	**—**	**231.8 USD**

The use of four independent frames slightly increased the total cost but enabled improved experimental accuracy and flexibility. Among the reflector options, mirror glass provided the highest optical gain at a modest cost, while galvanized steel offered the most balanced performance-to-cost ratio.

#### IoT-based data logger

The IoT-based data logger was designed to acquire simultaneous electrical and thermal measurements from all four PV modules. The system incorporated an ESP32 microcontroller, ACS712 Hall-effect current sensors, precision voltage dividers, DS18B20 temperature sensors, and regulated power electronics. Table 4 provides the full cost breakdown in USD.

**Table 4:** Estimated cost of the IoT-based data logger (USD).

Component	Specification	Qty	Unit Price (USD)	Total (USD)
ESP32 microcontroller	Dual-core, Wi-Fi enabled	1	7.5	7.5
ACS712-5 A current sensors	± 5 A measurement range	4	2.6	10.4
Voltage divider network	Precision resistors (± 1%)	4	0.65	2.6
DS18B20 temperature sensors	Digital, waterproof type	5	2.7	13.5
Power regulation and wiring	DC/DC converter set	1	5.4	5.4
Enclosure and connectors	Plastic housing	1	6.5	6.5
**Total Data Logger**	**—**	**—**	**—**	**45.9 USD**

The total cost of the IoT-based monitoring system was approximately 46 USD, representing an 87% reduction compared with commercial PV data loggers that typically exceed 370 USD. Despite its low cost, the system demonstrated high measurement accuracy, stability, and suitability for field deployment.

#### Techno-economic scalability analysis for commercial PV modules

To evaluate the practical scalability of the proposed reflector-enhanced configuration, a commercial utility-scale photovoltaic module was considered. A typical 550 W mono-PERC module (area ≈ 2.6 m², efficiency ≈ 21%) was selected as a representative case for modern grid-connected installations.

To maintain geometric consistency with the experimental setup, the reflector area was proportionally scaled to preserve an approximately 1:1 reflector-to-module area ratio. Based on the experimental cost analysis, the mirror reflector material corresponds to approximately 10.75 USD per square meter. Therefore, for a 2.6 m² commercial module, the estimated reflector material cost is approximately 28 USD per module.

The average cost of photovoltaic installations in Egypt typically ranges between 0.3 and 0.5 USD/Wp for small- to medium-scale systems, depending on system size, component quality, and installation conditions.

For a 10-kW system composed of approximately 18 modules (10,000 W ÷ 550 W ≈ 18), the total reflector material cost is estimated at approximately 504 USD. Including additional structural adaptation, mounting reinforcement, and installation overhead (estimated conservatively at 15%), the total reflector integration cost becomes approximately 580 USD for the entire 10 kW system.

Under Cairo climatic conditions, the average annual energy yield is approximately 1,700 kWh/ kWp^32,33^. Thus, a 10-kW system is expected to generate approximately 17,000 kWh annually. Based on the experimental results and adopting a conservative net performance enhancement of 15% (to account for scale-related thermal and optical losses), the additional annual energy yield would be approximately: 17,000 × 0.15 ≈ 2,550 kWh/year.

Assuming an electricity value of 0.10 USD/kWh, this corresponds to approximately 255 USD of additional annual revenue. Consequently, the simple payback period of the reflector integration would be approximately: 580 ÷ 255 ≈ 2.3 years.

The effectiveness of reflector-assisted PV systems strongly depends on the installation conditions and environmental factors. Reflectors are particularly beneficial in applications with limited installation area, where increasing the number of PV modules is not feasible. In such cases, they provide a practical solution to enhance energy yield without expanding the system footprint. While reflector-assisted PV systems are generally more effective in regions with high solar irradiance, their added value under such conditions should be carefully evaluated. In many high-irradiance regions, such as Egypt, PV systems already operate under near-optimal solar input, which may reduce the relative need for additional optical enhancement.

Moreover, the use of reflectors in hot climates introduces an additional thermal load on the PV modules. Since PV efficiency decreases with increasing temperature, the temperature rise induced by reflectors may partially offset the gains in irradiance. This effect becomes more significant during peak summer conditions, where module temperatures are already elevated.

Therefore, the practical benefit of reflectors depends on achieving a balance between optical gain and thermal losses. In some cases, especially where land availability is not a constraint, increasing the number of PV modules may be a more straightforward solution than using reflectors. However, reflector-assisted systems remain advantageous in space-constrained applications or where structural or economic limitations restrict system expansion.

### Discussion

#### Comparison with previous studies

The findings of this study are consistent with previous research, which has shown that reflectors can significantly increase the irradiance incident on PV panels, thereby improving power output. For instance^34^, reported that the use of flat mirror reflectors enhanced the overall power generation of the PV array improved by up to 57%, depending on the tilt angle and season. In the current study, similar trend improvements were observed, particularly when using mirror reflectors at 30°, which yielded the highest daily energy gains.

Other studies, such as^35^, highlighted the trade-off between improved irradiance and increased panel temperature. This trend was also evident in the present results, as higher reflector gains were accompanied by increased module temperatures, especially with mirror reflectors (+ 7 °C).

#### Impact of reflector materials

The results further demonstrate that the choice of reflector material has a direct impact on system performance. Mirrors, due to their high reflectivity, provided the highest energy enhancement but also induced greater heating effects. Galvanized sheet metal offered moderate reflectivity and heating, while aluminum foil showed the least improvement but is the most cost-effective. These findings suggest that material selection should balance energy yield with thermal management considerations, especially in hot climates such as Egypt.

#### Influence of reflector angle

The reflector inclination angle was found to be another critical parameter. At 30°, the reflectors achieved the best overall performance, confirming earlier reports^36^ that the optimal angle closely matches the PV panel tilt. At 20° and 40°, performance gains were smaller, likely due to performance reduction likely associated with suboptimal reflector–sun geometric interaction under the tested fixed configuration between the reflected rays and the panel surface.

#### Implications for practical applications

From a practical perspective, the results indicate that integrating simple, low-cost reflectors can provide a viable means of boosting PV energy generation in developing countries where resources are limited. However, the thermal penalty introduced by concentrated irradiance must be carefully managed, possibly by combining reflectors with cooling strategies such as passive heat sinks or active water cooling.

#### Limitations of the study

Despite the promising results, some limitations must be noted. The analysis was conducted during August under clear-sky conditions; seasonal variations and cloudy conditions were not considered. Furthermore, the experiments were limited to three reflector materials, while other surfaces (e.g., white-painted boards, polymers) could provide alternative solutions.

#### Contribution of the data logger

A significant contribution of this research lies in the development and application of the IoT-based data logger. Unlike previous studies that relied on commercial measurement systems, the proposed device enabled continuous, real-time monitoring of voltage, current, temperature, and irradiance. This approach not only reduced costs but also enhanced the flexibility of data acquisition for future PV research and applications.

## Conclusion and future work

This study presented the design and development of a low-cost IoT-based data logger for photovoltaic and environmental monitoring, alongside an experimental investigation of reflector-assisted PV systems. The developed data logger successfully measured and stored real-time parameters including voltage, current, power, irradiance, and panel/ambient temperature, providing reliable datasets for performance evaluation.

The experimental results demonstrated that using reflectors significantly improves PV performance. Among the tested reflector materials, mirror reflectors achieved the highest energy enhancement (up to ~ 21% at 30° inclination) but also led to the greatest increase in panel temperature. Galvanized sheet metal reflectors provided a balanced improvement, while aluminum foil reflectors offered a cost-effective but lower gain. The 30° inclination angle was identified as the optimal reflector configuration, yielding the best trade-off between irradiance gain and thermal penalty.

These findings confirm that low-cost reflectors can enhance solar energy generation, and that locally available materials provide an affordable pathway to improve PV system efficiency in resource-constrained environments such as Egypt.

Building upon the outcomes of this study, several directions are recommended to further advance the understanding and practical deployment of reflector-assisted photovoltaic systems. Future investigations should include seasonal performance assessments to evaluate reflector behavior under different solar paths and irradiance levels, ensuring comprehensive performance characterization throughout the year. Additionally, the long**-**term durability and reflectivity stability of materials such as mirrors, aluminum foils, and galvanized sheet metal should be examined under real outdoor conditions involving dust accumulation, humidity, and weathering effects. To address the thermal penalties observed during operation, future designs may integrate passive or active cooling mechanisms to mitigate temperature-induced efficiency losses. Further research could also explore advanced reflector geometries, including curved or compound surfaces, to optimize light redirection while minimizing thermal stress. On a broader scale, techno-economic assessments and life-cycle analyses are essential to evaluate the scalability and cost-effectiveness of such systems for commercial and rural applications. Finally, coupling the developed IoT-based data logger with machine learning algorithms offers a promising avenue for predictive modeling, real-time fault detection, and adaptive optimization of PV performance. Collectively, these efforts will contribute to the development of more efficient, low-cost, and sustainable solar energy technologies tailored to the needs of developing regions.

## Data Availability

The datasets used and/or analyzed during the current study available from the corresponding author on reasonable request.
